# The Type of Forage Substrate Preparation Included as Substrate in a RUSITEC System Affects the Ruminal Microbiota and Fermentation Characteristics

**DOI:** 10.3389/fmicb.2017.00704

**Published:** 2017-04-20

**Authors:** Andrea C. Duarte, Devin B. Holman, Trevor W. Alexander, Zoey Durmic, Philip E. Vercoe, Alexandre V. Chaves

**Affiliations:** ^1^Faculty of Veterinary Science, School of Life and Environmental Sciences, The University of Sydney, SydneyNSW, Australia; ^2^Lethbridge Research Centre, Agriculture and Agri-Food Canada, LethbridgeAB, Canada; ^3^The University of Western Australia, School of Agriculture and Environment, CrawleyWA, Australia

**Keywords:** RUSITEC, microbial ecology, rumen, methane, fermentation, forage, microbiota, cattle

## Abstract

*In vitro* fermentation systems such as the rumen simulation technique (RUSITEC) are frequently used to assess dietary manipulations in livestock, thereby limiting the use of live animals. Despite being in use for nearly 40 years, improvements are continually sought in these systems to better reflect and mimic natural processes in ruminants. The aim of this study was to evaluate the effect of forage preparation, i.e., frozen minced (FM) and freeze-dried and ground (FDG), on the ruminal microbiota and on fermentation characteristics when included as a substrate in a RUSITEC system. A completely randomized design experiment was performed over a 15-day period, with 7 days of adaptation and an 8-day experimental period. Fermentation parameters (total gas, CH_4_, and volatile fatty acid production) were analyzed on a daily basis over the experimental period and the archaeal and bacterial microbiota (liquid-associated microbes [LAM] and solid-associated microbes [SAM] was assessed at 0, 5, 10, and 15 days using high-throughput sequencing of the 16S rRNA gene. Results from this study suggested a tendency (*P* = 0.09) of FM treatment to increase daily CH_4_ (mg/d) production by 16.7% when compared with FDG treatment. Of the major volatile fatty acids (acetate, propionate, and butyrate), only butyrate production was greater (*P =* 0.01) with FM treatment compared with FDG substrate. The archaeal and bacterial diversity and richness did not differ between the forage preparations, although feed particle size of the forage had a significant effect on microbial community structure in the SAM and LAM samples. The *Bacteroidetes* phylum was more relatively abundant in the FM substrate treatment, while *Proteobacteria* was enriched in the FDG treatment. At the genus-level, *Butyrivibrio, Prevotella*, and *Roseburia* were enriched in the FM substrate treatment and *Campylobacter* and *Lactobacillus* in the FDG substrate treatment. Evidence from this study suggests that forage preparation affects CH_4_ production, butyrate production, and the structure of the rumen microbiota during *in vitro* fermentation.

## Introduction

Reducing methane (CH_4_) emissions from anthropogenic activities is of considerable interest since enteric fermentation from ruminants accounts for 25% of the 40% derived from agriculture (Olivier et al., 1999; Steinfeld et al., 2006). Ruminants are considered economically important due to their capacity to digest low-quality forages (Flint, 1997) and their ability to convert these substrates into energy is largely dependent on the rumen microbiota (i.e., bacteria, anaerobic fungi, protozoa, and methanogenic archaea) which converts indigestible plant material into usable energy for the host. In addition, this allows ruminants to produce milk, meat, wool, and leather without competing directly with humans for food (Buddle et al., 2011).

Rumen microbial communities are known to respond to changes in diet, environment and to a lesser extent, the type of host (Henderson et al., 2015). Diet type (concentrate, forage), as well as the preparation, influences the fermentation process and the composition of the rumen microbiota (Henderson et al., 2015). *In vitro* fermentation systems such as the rumen simulation technique (RUSITEC) are frequently used to assess dietary manipulations in livestock, reducing the use of live animals for experiments. Although the RUSITEC system has been used in research for almost 40 years (Czerkawski and Breckenridge, 1977), improvements are sought in these systems that may better reflect and mimic natural processes in ruminants. For example, there is a requirement for appropriate methods that describe the preparation of fresh forage material to be used in *in vitro* systems. In recent years, it has been common practice to use forages in freeze-dried and ground (FDG) form as a substrate in these systems (Avila-Stagno et al., 2014), however, digestion products and kinetics are altered when fresh forage is used (Barrell et al., 2000).

The type of feed preparation has very significant effects on degradation kinetics, in terms of constituent disappearance, proteolysis, volatile fatty acids production, and microbial growth. Although conventional feed preparation involves freeze drying and grinding, this is not appropriate for fresh forages which have been both minced and freeze dried for comparison (McNabb et al., 1996). Barrell et al. (2000) and Cohen and Doyle (2001) demonstrated degradation kinetics of fresh chopped, minced and also freeze dried and ground material. They suggested the minced preparation to be most appropriate for fresh grasses and legumes. *In vitro* incubations should be carried out with feed prepared in a way that best mimics chewing by ruminants.

Presently, limited research has been conducted on the effect of the forage/substrate form on the rumen microbiota or on fermentation parameters in *in vitro* systems. The aim of this study was to evaluate the effect of two different forms of forage substrate, frozen minced (FM) and FDG, on fermentation parameters (including CH_4_) and the archaeal and bacterial microbiota, using a RUSITEC system. Our hypothesis was that by changing substrate preparation, digestibility, fermentation characteristics, and microbial population would be severely impacted.

## Materials and Methods

### Experimental Design and Treatments

This study was conducted using a RUSITEC system containing six fermentation vessels with samples taken over a 15-day period (7 days of adaptation followed by 8 days where samples were taken). The experiment was a completely randomized design with two treatments and three replicates per treatment. The fermentation substrate consisted of equal portions of concentrate and ryegrass that was included either as FM preparation (FM treatment) or FDG to 2 mm forage (FDG treatment). Fermentation parameters (total gas, CH_4_, and volatile fatty acid [VFA] production) were analyzed on a daily basis over the experimental period and the archaeal and bacterial microbiota (liquid-associated microbes [LAM] and solid-associated microbes [SAM]) were characterized on days 0, 5, 10, and 15.

### Plant Material

Plant material of ryegrass (*Lolium perenne*) was collected on 15th January 2015 at the May Farm research site of the University of Sydney, Camden Campus, NSW, Australia (34°04′ S; 150°81 69′ E). The climate is warm-temperate with a mean annual minimum and maximum temperature of 10.7 and 23.3°C, respectively. The annual average rainfall is 738 mm (1900–2010). Multiple samples were randomly selected and harvested at grazing height ≥ 5 cm above ground level to mimic grazing by cattle. Plant material was processed immediately upon return to the laboratory (within 45 min of collection) and divided into two equal portions. One half of the plant material (FM sample) was prepared according to the methodology described by Chaves et al. (2006) as follows: material was frozen at -20°C and while still frozen, cut into 2–3 cm lengths using scissors and minced using a meat mincer (Rovtek MG-22SS, Commercial Meat Mincer, 800 W, 250 kg/h, blade speed 190 rotation/h, Sydney, NSW, Australia) fitted with a screen plate with 12 mm holes. The samples were stored at -20°C until the day of incubation. Preparation of FDG samples involved freeze-drying the ryegrass, grinding and then passing the ground ryegrass through a 2 mm screen. Samples were kept at room temperature until the day of incubation.

### Chemical Analysis

Ryegrass and concentrate were analyzed for dry matter (DM) (method 967.03), ash (method 942), and ether extract (EE) content by extraction with diethyl ether using an Ankom XT10 Extraction System (Ankom^®^ Technol. Corp., Fairport, NY, USA; method 920.39), following Association of Official Analytical Chemists [AOAC] (2006) methods. The concentration of neutral detergent fiber (NDF) was determined using procedures detailed by Van Soest et al. (1991) and modified for an Ankom 200/220 Fiber Analyzer (Ankom Technol. Corp., Fairport, NY, USA) using sodium sulfite and heat stable α-amylase. Crude Protein (CP) was analyzed by combustion [method 990.03 (Association of Official Analytical Chemists [AOAC], 2006)] using FP628 Food/Protein Analyzer (LECO, St Joseph, MI, USA) following the manufacturer’s guidelines. The feed chemical composition is presented in Supplementary Table S1.

### Substrate and Rumen Inoculum

The rumen inoculum was collected on the initial day of the experiment from one ruminally fistulated (Soliva et al., 2011, 2015) Holstein dairy cow (9 years old, 750 kg). The donor animal was housed at The University of Sydney Corstorphine Dairy farm and was cared for in accordance with the guidelines of The University of Sydney Animal Ethics Committee (Project number 2015/835). The cow was fed the same diet (Supplementary Table S1) that was used as a substrate in the RUSITEC fermenters and the inoculum was collected 2 h after the morning feeding. The rumen digesta was filtered through four layers of cheesecloth to separate the solid and liquid portion to be used as an initial inoculum. The rumen inoculum was transported immediately to the laboratory using insulated containers.

Approximately 5 g (DM basis) of each of the treatments was weighed into pre-weighed nylon bags (70 mm × 140 mm; pore size = 150 μm) and approximately 5 g (DM basis) of concentrate feed was weighed into a separate nylon bag (70 mm × 100 mm; pore size = 150 μm). At the start of the experiment, 4.7 L of rumen fluid was evenly distributed into six fermentation vessels, and equal amounts (i.e., ∼50 g) of rumen solids were weighed into nylon bags.

### RUSITEC Fermentation

The RUSITEC apparatus was equipped with six 800 mL fermentation vessels. Each vessel had an inlet for the infusion of buffer and an effluent output port. At the start of the experiment each fermentation vessel was filled with 780 mL of rumen fluid. The nylon bags (containing about 30 g of wet weight of rumen solids and the experimental diets) were placed inside each fermenter according to the randomized treatments. Fermenters were then submerged in a 39°C water bath and infused with McDougall’s buffer [3.69 g/L Na_2_HPO_4_ (anhydrous), NaHCO_3_ 9.8 g/L, NaCl 0.47 g/L, KCl 0.57 g/L, MgCl_2_6H_2_O 0.061 g/L, CaCl_2_2H_2_O 0.0336 g/L] at a dilution rate of 30 mL/min.

After 24 h of incubation, the rumen solids bag was replaced with new nylon bags containing the experimental treatments, and from day 2 onward, the nylon bags were replaced each day, meaning that four bags were present at any given time (Narvaez et al., 2013; Avila-Stagno et al., 2014).

### Total Gas and Methane Production

Total gas produced was collected on a daily basis in gas-tight bags (Plastigas, Linde AG, Munchen, Germany) connected to the effluent flasks. The total volume of gas was determined by water displacement. The gas bag was connected to a flask filled with water and the gas was evacuated by applying manual pressure to the flask. The evacuated water was then collected in a graduated cylinder and the volume collected was measured as daily gas production and expressed in mL/d.

From day 8 until the end of the experiment, 15 mL from each gas bag was removed with a syringe and transferred to an exetainer tube (Labco Ltd, Lampeter, UK) for analysis of methane concentration by gas chromatography (Bruker 450 GC, Bruker Technologies, Australia, with two packed columns and a Compass CDS data acquisition software (Bruker Technologies, Australia). Argon and helium were used as carrier gases at a flow rate of 30 mL/min. The oven, injector and detector temperatures were 50, 70, and 180°C, respectively.

### Dry Matter Disappearance

Dry matter disappearance was determined using the residue remaining in each nylon bag after 48 h of fermentation. Nylon bags were removed, washed with cold water (tap water/distilled water) until the water was clear (Narvaez et al., 2013; Li et al., 2014) and then dried at 100°C for 24 h. The residue weight was recorded and used for the calculation of DM disappearance.

### Fermentation Parameters and Volatile Fatty Acid (VFA) Production

Fermentation parameters, such as pH and volume outflow, were determined on a daily basis during the 15-day experimental period (i.e., 7 days of adaptation and 8 days of collection). Volume was measured daily at the time of feed bag exchange with a graduated cylinder. Starting at day 8, individual samples from each fermentation vessel were taken for quantification of VFA. Samples were preserved with metaphosphoric acid (25% w/v; 1:5 dilution) and stored at -20°C until analysis. Capillary gas chromatography was performed to analyze VFA (C:2 to C:6) according to the Biochemistry Laboratory Method NTR-7, Issue No. 5, 05/05/2015 (University of Western Australia). An Agilent 6892 Series GC with Agilent 7696 sample preparation station and HP 6890 injector with HP Chemstation software were used. The capillary column was a HP-FFAP, 30 m × 0.53 mm × 1.0 μm (HP Part. No. 199095F-123). Oven temperature was 240°C and hydrogen was used as carrier with a total flow of 55.7 mL/min at 3.5 psi. The HP Chemstation system calculated the concentration for the VFA in mmol/L. Concentration of volatile fatty acids production was calculated per day by multiplying concentration and volume of outflow.

### Sample Collection for Microbiota Analysis

In order to investigate the effect of forage preparation on the rumen microbial community, samples were taken at the start of the experiment (day 0) from the original rumen fluid and solids, and on days 5, 10, and 15 for rumen fluid from the RUSITEC fermenters. To characterize LAM, a 10 mL sample from each fermenter was collected into a sterilized 25 mL falcon tube, immediately frozen in liquid nitrogen and kept at -80°C until DNA extraction. To assess SAM, on the last day of the experiment (i.e., day 15), the digesta in the nylon bags was frozen in liquid nitrogen and then subjected to freeze drying before DNA extraction.

### DNA Extraction and Illumina Sequencing of the Archaeal and Bacterial 16S rRNA Gene

Total DNA was extracted using a QIAamp Fast DNA stool mini kit (Qiagen), according to manufacturer’s instructions and a lysis temperature of 80°C. The DNA yield and purity was assessed using a NanoDrop 2000 Spectrophotometer (Thermo Scientific) and extracted DNA was stored at -20°C. The 16S rRNA gene libraries were generated using a two-step PCR protocol. The first PCR step amplified the V4 region of the 16S rRNA gene using the universal bacterial and archaeal primers 515-F (5′-GTGCCAGCMGCCGCGGTAA-3′) and 806-R (5′-GGACTACVSGGGTATCTAAT-3′) (Caporaso et al., 2011). The second PCR step was used to add a unique 10-bp barcode at the 5′ end of the amplicon as well to add Illumina adapters. All PCR amplification and sequencing steps were carried out at Genome Quebec (Montreal, QC, Canada). Briefly, the 16S rRNA gene amplicons were quantified using a Quant-iT PicoGreen dsDNA assay kit (Invitrogen, Burlington, ON, Canada), pooled in equimolar ratios, and then purified with AMPure XP beads (Beckman Coulter, Mississauga, ON, Canada). The 16S rRNA gene amplicons were then sequenced using an Illumina MiSeq (2 × 250) and the MiSeq Reagent Kit v2 (500 cycles; Illumina, San Diego, CA, USA) according to manufacturer’s instructions.

### 16S rRNA Gene Sequence Analysis

The 16S rRNA gene sequences were processed and analyzed within the QIIME software package v. 1.9.1 (Caporaso et al., 2010b). Paired-end reads were joined using fastq-join with a minimum overlap of 35 bp and a maximum percent difference of 15 (Aronesty, 2013). Joined sequences were quality filtered with sequences being truncated following three consecutive base calls of a Phred score of less than 25. Sequences were retained only when 75% or more of the original sequence remained after truncation. Chimeric sequences were removed using the UCHIME algorithm (Edgar et al., 2011) implemented in USEARCH v. 6.1544 (Edgar, 2010). Sequences were then clustered into operational taxonomic units (OTUs) at 97% similarity using an open reference OTU picking method and the SILVA database v. 111 (Pruesse et al., 2007). Sequences that did not match OTUs in the SILVA database were clustered into OTUs using the *de novo* approach and USEARCH. The UCLUST consensus taxonomy assigner (Edgar, 2010) was used to assign taxonomy to OTUs using the SILVA database, with a minimum similarity of 0.8 and max accepts of 3. Representative sequences for the OTUs were aligned using PyNast (Caporaso et al., 2010a) and a phylogenetic tree was created using FastTree (Price et al., 2010). OTUs containing fewer than 10 sequences were excluded from further analysis as well as those OTUs classified as chloroplasts.

Each sample was randomly subsampled to 22,500 sequences per sample to account for uneven sequencing depth. The bacterial and archaeal diversity in each sample was calculated within QIIME using the Shannon index (Shannon, 1948) and phylogenetic diversity (PD whole tree) (Faith, 1992). Unweighted UniFrac distances (Lozupone and Knight, 2005) were used to assess the archaeal and bacterial community structure (beta-diversity) of each forage preparation type, sampling time, and sample type (solid vs. liquid). The subsequent distance matrices were visualized as principal coordinate analysis (PCoA) plots using Emperor (Vazquez-Baeza et al., 2013).

All 16S rRNA gene sequences were submitted to the NCBI Sequence Read Archive (SRA) under bio-project accession PRJNA304765^1^.

### Statistical Analysis

Fermentation and alpha-diversity data were analyzed using the MIXED procedure of SAS (SAS, Inc., 2015; SAS Online Doc 9.1.3). The model included the fixed effects of forage preparation, day and forage preparation × day interaction. Therefore, the individual fermenter was used as the experimental unit for statistical analysis. The minimum values of Akaike’s information criterion were used to select the covariance structure. Significance was declared at *P* ≤ 0.05 and a trend was discussed when 0.05 < *P* ≤ 0.10.

Unweighted UniFrac distances were compared using ANOSIM (analysis of similarities) with 999 permutations. OTUs that were differentially abundant by sample type, forage preparation type and sampling time were identified using the *G*-test of independence with a false discovery rate (FDR) < 0.05. Linear discriminant analysis effect size (LEfSe) was used to determine which phyla and genera were enriched at each sampling time, forage preparation type, and sample type. LEfSe uses the Kruskal–Wallis test to identify different (*P* < 0.05) genera among sample groups and uses linear discriminant analysis (LDA) to estimate the effect size of each of these (Segata et al., 2011). A LDA score of 3.5 and an overall relative abundance of greater than 0.01% was used as the threshold for identifying differentially abundant genera.

## Results

### Fermentation Parameters

**FIGURE 1:**
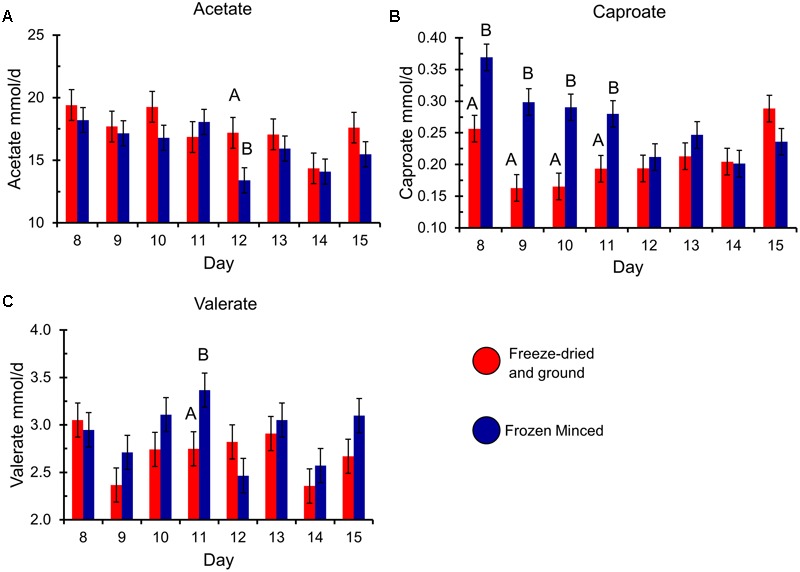
**Production of the volatile fatty acids (A)** acetate, **(B)** caproate, and **(C)** valerate, by experimental day and forage preparation type. Error bars represent standard error of the mean. Forage preparation types that significantly differ (*P* < 0.05) within each experimental day are indicated by different uppercase letters.

The type of forage substrate preparation did not affect total gas, CH_4_ (%, mg/g DM, and mg/g DDM) or digestibility of substrates (*P ≥* 0.13; **Table 1**). Total VFA, propionate, BCVFA (branched-chain volatile fatty acids), acetate to propionate ratio and pH were also not affected by type of preparation, but there was a tendency of increase in daily CH_4_ (mg/d) production in FM compared to FDG (**Table 2**). An interaction between forage preparation and sampling time was observed for acetate, valerate and caproate (**Figure 1**; *P* ≤ 0.05). Butyrate production was greater (**Table 1**; *P* = 0.01) in FM compared to FDG treatments.

**Table 1:** Effects of forage preparation on total gas, methane, and substrate disappearance in the RUSITEC fermentation.

				*P*-value		
	Freeze-dried and ground	Frozen minced	SEM	Preparation	Day	Preparation × Day
Total gas, mL/d	1708.5	1758.6	144.59	0.82	0.05	0.66
Total gas, mL/g DM	170.0	179.0	10.13	0.55	<0.01	0.21
Total gas, mL/g DMD	193.6	204.0	11.54	0.55	<0.01	0.23
CH4, %	7.3	7.4	0.46	0.88	0.44	0.85
CH4, mg/d	90.8	106.0	5.54	0.09	0.10	0.46
CH4, mg/g DM	9.0	10.3	0.54	0.13	0.10	0.47
CH4, mg/g DMD	10.3	11.7	0.68	0.20	0.15	0.63
Forage IVDMD, %	91.1	92.3	0.67	0.24	0.02	0.12
Concentrate IVDMD, %	84.5	83.2	0.64	0.19	0.07	0.02

*SEM, standard error of mean; DM, dry matter; DMD, dry matter disappearance; IVDMD, *in vitro* dry matter disappearance.*

**Table 2:** Effect of forage preparation on pH and volatile fatty acids (VFA) production.

				*P*-value		
	Freeze-dried and ground	Frozen minced	SEM	Preparation	Day	Preparation × Day
pH	6.9	6.9	0.005	0.82	0.06	0.56
Total VFA, mmol/d	37.2	36.3	0.83	0.51	<0.01	0.21
Acetate, mmol/d	17.4	16.1	0.41	0.07	<0.01	0.05
Propionate, mmol/d	9.5	8.9	0.25	0.17	0.03	0.19
Butyrate, mmol/d	5.3b	6.0a	0.16	0.01	0.01	0.19
Valerate, mmol/d	2.7	2.9	0.06	0.05	0.27	0.03
Caproate, mmol/d	0.2b	0.3a	0.01	0.02	<0.01	0.01
BCVFA, mmol/d	2.1	2.1	0.06	0.51	0.02	0.64
Acetate:propionate	1.8	1.8	0.05	0.71	<0.01	0.42

*Different lowercase letters indicate significant differences between freeze-dried and ground and frozen minced prepared forages (*P* < 0.05). BCVFA, branched-chain VFA; iso-butyrate + iso-valerate.*

### Archaeal and Bacterial Microbiota

**FIGURE 2:**
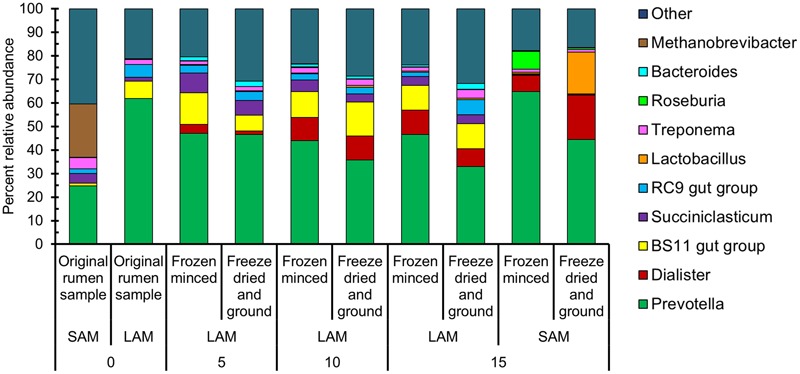
**The 10 most relatively abundant genera among all samples for each sample type, forage preparation type, and sampling day.** LAM, liquid-associated microbes; SAM, solid-associated microbes.

A total of 858,881 sequences with an average length of 262 bp among all samples remained following quality filtering and removal of primer sequences. These sequences were clustered into 3,349 OTUs. Overall, the most relatively abundant phyla were: *Bacteroidetes* (60.1%), *Firmicutes* (24.3%), *Spirochaetes* (3.8%), *Euryarchaeota* (3.7%), *Planctomycetes* (2.6%), *and Proteobacteria* (1.6%) (**Supplementary Figure S1**). At the genus-level, *Prevotella* was the most relatively abundant (44.8%), while *Dialister* (8.4%), *Succiniclasticum* (3.5%), *Lactobacillus* (2.6%), and *Treponema* (2.1%) were the only other genera with an overall relative abundance > 2.0% (**Figure 2**).

In regards to methanogens, only the solid fraction from the original rumen sample had a relatively high abundance of methanogens (classes *Methanobacteria* and *Methanomicrobia*; 22.9%). Nearly all of these methanogenic sequences were identified as *Methanobrevibacter* (22.6%). Excluding the original rumen samples, the relative abundance of the methanogens was 1.6% among all samples. Apart from *Methanobrevibacter*, the three other methanogenic genera identified among all samples were *Methanosphaera, Methanomicrobium*, and *Methanimicrococcus*. Overall, there were 28 OTUs that were classified into one of the four genera above.

Alpha-diversity (within-sample diversity) measures are presented in **Table 3** for each sample preparation type. Archaeal and bacterial diversity and richness were not affected by forage preparation type (*P* > 0.05) in LAM samples, however, lower phylogenetic diversity was observed in LAM samples at day 5 compared with days 10 and 15 (68.84 vs. 74.8 and 74.2, respectively; *P* < 0.05). The archaeal and bacterial diversity and richness in SAM samples were also not affected by the forage preparation (*P* > 0.05; **Table 3**).

**Table 3:** Archaeal and bacterial richness and diversity measures for liquid-associated microbes (LAM) and solid-associated microbes (SAM) samples for freeze-dried and ground and frozen minced forage types.

				*P*-value		
	Freeze-dried and ground	Frozen minced	SEM	Preparation	Day	Preparation × Day
**LAM**						
Shannon index	4.34	4.16	0.105	0.257	0.831	0.233
PD whole tree	72.2	73.1	1.06	0.541	0.022	0.172
Number of OTUs	1070	1088	26.0	0.643	0.243	0.135
**SAM**						
Shannon index	3.61	3.87	0.101	0.141	–	–
PD whole tree	59.7	59.1	2.404	0.848	–	–
Number of OTUs	908	947	49.445	0.607	–	–

*PD, phylogenetic diversity; OTU, operational taxonomic unit.*

**FIGURE 3:**
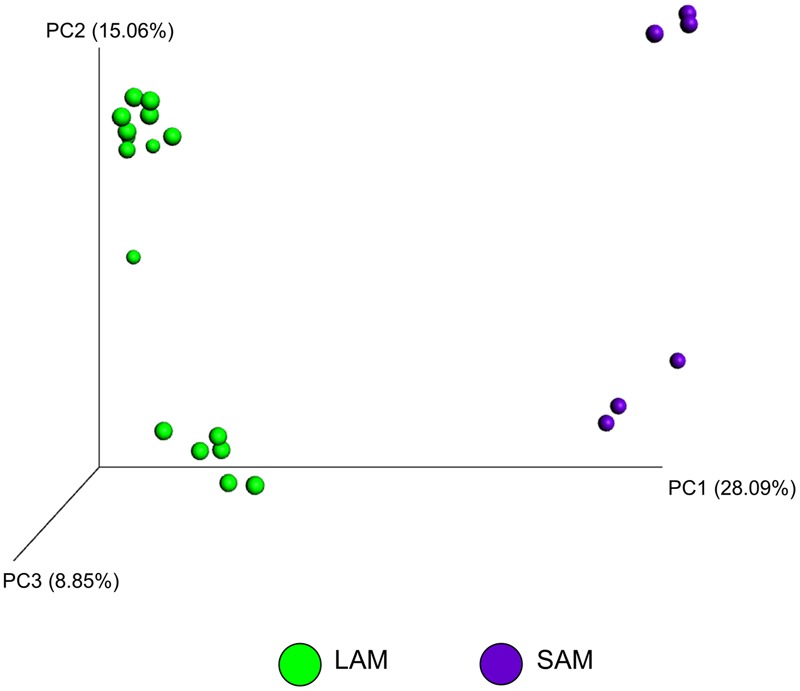
**Principal coordinate analysis (PCoA) of the unweighted UniFrac distances for SAM and LAM samples.** The percent variation explained by each principal coordinate is indicated on the axes. Original rumen samples are excluded. LAM, liquid-associated microbes; SAM, solid-associated microbes.

**FIGURE 4:**
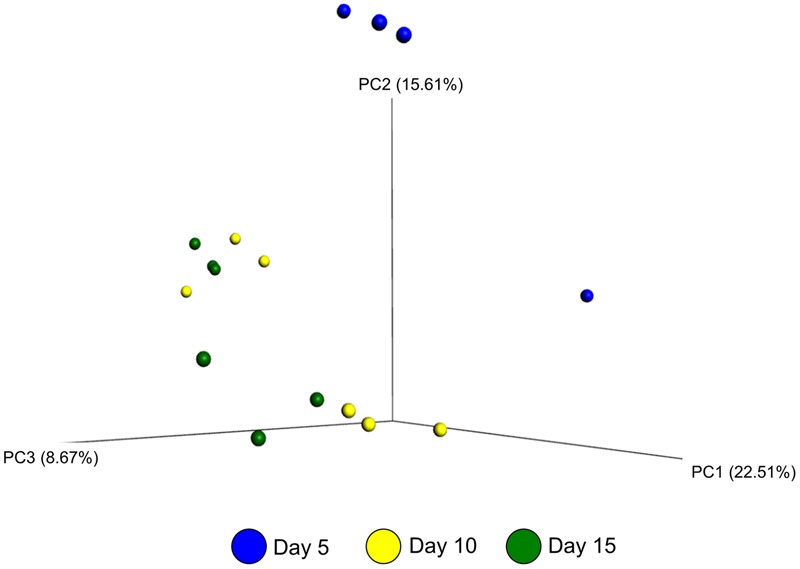
**Principal coordinate analysis of the unweighted UniFrac distances for LAM samples at different sampling days.** The percent variation explained by each principal coordinate is indicated on the axes. Original rumen samples are excluded. LAM, liquid-associated microbes.

**FIGURE 5:**
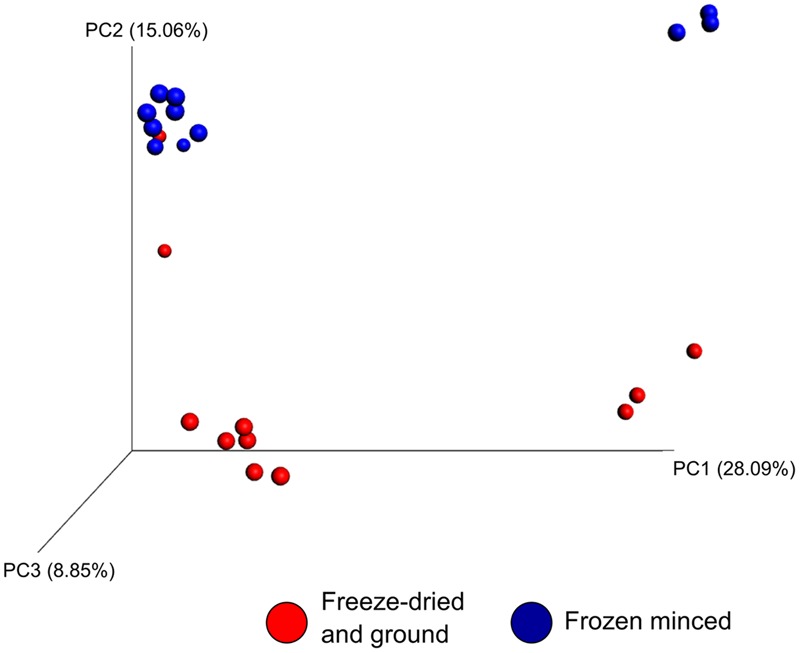
**Principal coordinate analysis of the unweighted UniFrac distances for forage preparation for both LAM and SAM samples.** The percent variation explained by each principal coordinate is indicated on the axes. No rumen samples included LAM, liquid-associated microbes; SAM, solid-associated microbes.

The effect of microbial sample type (LAM vs. SAM), forage preparation, and sampling time, on the structure of the archaeal and bacterial microbiota was analyzed using unweighted UniFrac distances (**Figures 3–5**). Visual inspection of the PCoA plots and ANOSIM of unweighted UniFrac distances demonstrated that sample type (LAM vs. SAM) was the most important factor in determining the structure of the microbiota (**Figure 3**, *R*-value = 0.982; *P* = 0.001). Similar to the within-sample diversity analysis, sampling time also had a significant effect on the structure of the microbiota in LAM samples. In particular, samples from day 5 clustered separately from those of days 10 and 15 (**Figure 4**, *R*-value = 0.423; *P* = 0.005). Samples also clustered together based on forage preparation type (**Figure 5**, *R*-value = 0.261; *P* = 0.002).

**FIGURE 6:**
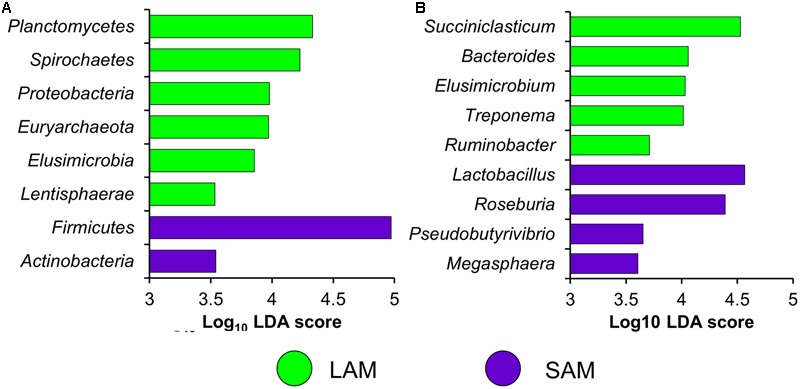
**Differentially abundant (A)** phyla and **(B)** genera between LAM and SAM samples as determined using linear discriminant analysis effect size (LEfSe) analysis. Only phyla and genera with a LDA score >3.5 and an overall relative abundance of >0.01% are included.

Differentially abundant OTUs were identified using the *G*-test of independence. Excluding the original rumen samples, there were 244 OTUs that were differentially abundant (FDR < 0.05) between sample type (LAM vs. SAM) and 118 differentially abundant OTUs between the two forage preparations (both SAM and LAM samples included). Phyla enriched in one of the two sample types (LAM vs. SAM) were identified using LEfSe (**Figure 6A**; LDA [log_10_] score > 3.5). There was a total of six phyla enriched in the LAM samples and two in the SAM samples, with *Firmicutes* notably more relatively abundant in the SAM samples. At the genus-level, *Succiniclasticum, Bacteroides*, and *Treponema* were among the genera that were more relatively abundant in the LAM samples, while *Lactobacillus* and *Roseburia* were enriched in the SAM samples (**Figure 6B**).

Only the *Proteobacteria* phylum was more relatively abundant in the FDG samples and *Bacteroidetes* was the only phylum enriched in the FM samples (LDA [log_10_] score > 4.0; *P* < 0.05). In the FDG samples, the genera *Campylobacter* and *Lactobacillus* were more relatively abundant while *Butyrivibrio, Prevotella*, and *Roseburia* were more relatively abundant in the FM samples (LDA [log_10_] score > 3.5; *P* < 0.05). Interestingly, *Campylobacter* was almost completely absent from the FM samples, as 9 of the 11 samples analyzed had no *Campylobacter* sequences, while all but two of the 11 FDG samples had greater than 0.1%. Among sampling times in the LAM samples, *Firmicutes* was also enriched at day 10 and *Planctomycetes, Synergistetes, and Verrucomicrobia* more relatively abundant at day 15. No phyla were enriched at day 5 compared to the other two sampling times. Similarly, among the genera with an overall relatively abundance greater than 0.01%, only *Dialister* was enriched at day 10 and *Acidaminococcus* and *Megasphaera* in day 15 samples (LDA [log_10_] score > 4.0; *P* < 0.05).

## Discussion

Understanding and characterizing the rumen microbiota has become increasingly important in recent years as it has been demonstrated that the ruminal microbiota is influenced by and responds to changes in diet (Dehority, 2003; Henderson et al., 2015). In the present study, we examined the effect of FM vs. FDG forage preparation on CH_4,_ total gas, and VFA production, and on the archaeal and bacterial microbiota, using a RUSITEC system.

Our findings indicated that forage substrate preparation had very little effect on fermentation parameters. FM preparation increased (*P* = 0.10) methane production (mg/d) by 16% compared to FDG. This is in agreement with previous reports from *in vitro* studies where authors had used freeze-dried plant samples for assessing anti-methanogenic compounds or alternative feed sources (García-González et al., 2008; Durmic et al., 2010; Li et al., 2014). However, care must be taken when interpreting these results, as some trends were observed in terms of methane production, and it may be possible that the freeze-drying is underestimating methane production. This is not unexpected, as grinding reduces particle size which in turn may result in better digestion (McAllister et al., 1994) and less methane production (Hales et al., 2012). However, FM preparation of forages achieves a forage particle distritution similar to what occurs in the chewing activity McNabb et al. (1996) and Barrell et al. (2000).

In terms of the molar proportion of VFA, a significant increase in butyrate production for FM forage preparation was found, a result that is in agreement with Mohammed et al. (2014) who reported a greater proportion of butyrate in fresh forage compared with conserved forage. However, our results did not show the same increasing trend for acetate, even though there was an interaction between time and forage preparation (**Figure 1A**). This interaction was only observed on day 12 of the experiment. Since propionate production and the acetate:propionate ratio was not affected by fresh or dried forage, differences in the VFA molar proportions could be related to the shifts in microbial communities. As Mohammed et al. (2014) reported, fresh or dried forage could result in differences based on the availability of specific nutrients.

The archaeal and bacterial microbiota was significantly affected by both microbial sample type (LAM vs. SAM) and the type of forage substrate preparation. Although archaeal and bacterial diversity and richness were unchanged, there were differences in various taxa between the two treatments (**Figure 6**). The changes observed in the microbial community structure (**Figure 5**) are noteworthy because to our knowledge, this is one of the first studies to use high-throughput sequencing to evaluate the rumen microbiota in response to different forage preparations. Although a number of studies have been conducted to evaluate the effect of diet on the rumen microbial community structure, most of these have investigated the effect of total mixed ration diets (Jami et al., 2014; Pitta et al., 2014; Veneman et al., 2015) or type of forages (Huws et al., 2010), rather than the forage preparation type.

de Menezes et al. (2011) used pyrosequencing of the 16S rRNA gene to compare the effect of feed pasture vs. total mixed ration diets on the rumen microbiota, noting several changes in the bacterial microbiota between diets and between the liquid and solid content. Our results are consistent with previous findings, as we found that the rumen microbiota was significantly different between the liquid and solid fractions, as demonstrated through the phylogenetic-based unweighted UniFrac distances (**Figure 3**). Mohammed et al. (2014) also found that bacterial communities clustered primarily by rumen digesta phase (solid vs. liquid) and that the microbiota of each fraction was dissimilar from the other.

In terms of archaeal community structure, the low relative abundance of the phylum *Euryarchaeota* limited our ability to perform an analysis based only on the methanogens. Methanogens were only present at a relative abundance of greater than 5.2% in the original rumen inoculum (SAM). Nonetheless, we found that *Euryarchaeota*, excluding the original rumen inoculum, was enriched in the LAM samples (**Figure 6A**). These findings indicate that methane production in the rumen may be influenced by the relative abundance of Archaea, rather than the microbial population structure as argued by Wallace et al. (2014) and Veneman et al. (2015), since the FM forage preparation showed an increased trend on methane production.

In the current study, the rumen microbiota was dominated by the *Bacteroidetes* and *Firmicutes* phyla, in both FM and freeze-dried preparation samples. Similarly, de Menezes et al. (2011) reported that *Bacteroidetes* and *Firmicutes* are the most relatively abundant bacterial phyla (>80%) in the rumen of dairy cattle on pasture and concentrate diets. However, these authors also noted a relatively high abundance of *Fibrobacteres* sequences in the solid phase of TMR and pasture diets (5–10%), although in the present study it only accounted for less than 1% of the sequences among all samples. However, this result to similar to a study of the rumen microbiota by Jami and Mizrahi (2012) where the relative abundance of *Fibrobacteres* was 0.02% in the rumen of dairy cattle fed 30% roughage and 70% concentrate.

We observed that *Prevotella* was the dominant genus among all samples, a result similar to that of *in vivo* studies of the rumen microbiota (Jami and Mizrahi, 2012; Jami et al., 2013; Thoetkiattikul et al., 2013; Paz et al., 2016). Jami et al. (2014) reported that certain genera in the *Firmicutes* phylum such as *Dialister* and *Lactobacillus*, were also negatively correlated with *Prevotella.* In the current study, *Prevotella* was more relatively abundant in the FM samples and *Lactobacillus* in the FDG samples. The enrichment of *Bacteroidetes* in the FM forage and *Firmicutes* in the SAM samples concurs with the findings of de Menezes et al. (2011) where a pasture diet vs. TMR diet was compared *in vivo*. In terms of forage preparation, the fact that *Butyrivibrio, Prevotella*, and *Roseburia* were enriched in the FM samples is consistent with the increased concentration of butyrate in these samples, as all of these genera contain major producers of butyrate (Flint et al., 2012; Sun et al., 2015). Caproate, the concentration of which was also greater (*P* < 0.05) in the FM samples, has also been found to be positively associated with *Prevotella* spp. (Tap et al., 2015). Therefore, the higher relative abundance of *Prevotella* in the FM samples may indicate that this forage preparation method results in a microbiota that is more similar to the natural rumen.

## Conclusion

We can partially reject our initial hypothesis since this study demonstrated that fermentation parameters are generally not affected by the type of forage preparation with the exception of butyrate and a small trend in methane production. *Prevotella* and the butyrate producers, *Butyrivibrio* and *Roseburia*, were more relatively abundant in the FM prepared forage treatment, suggesting that this forage preparation method yields a microbiota that is more similar to that of the natural rumen. The diversity and richness of the microbiota was also not influenced by forage treatment, however, the structure of the rumen microbiota was significantly different between the LAM and SAM samples, as well as between forage substrate treatments.

## Author Contributions

AD and AC designed the study. AD, AC, DH, TA, and ZD conducted the experimental procedures and laboratory analyses. AD, AC, DH, and TA analyzed and interpreted the data. AD drafted the manuscript. AD, AC, DH, TA, ZD, and PV critically revised the manuscript.

## Conflict of Interest Statement

The authors declare that the research was conducted in the absence of any commercial or financial relationships that could be construed as a potential conflict of interest.

## Footnotes

1 http://www.ncbi.nlm.nih.gov/sra

## Supplementary Material

FIGURE S1 **The eight most relatively abundant phyla among all samples for each sample type, forage preparation type, and sampling day.** LAM, liquid-associated microbes; SAM, solid-associated microbes.
