# Supplementary figures and images for: The Type of Forage Substrate Preparation Included as Substrate in a RUSITEC System Affects the Ruminal Microbiota and Fermentation Characteristics
Source: Front Microbiol. 2017 Apr 20;8:704. doi: 10.3389/fmicb.2017.00704 (PMC5397515; doi:10.3389/fmicb.2017.00704)

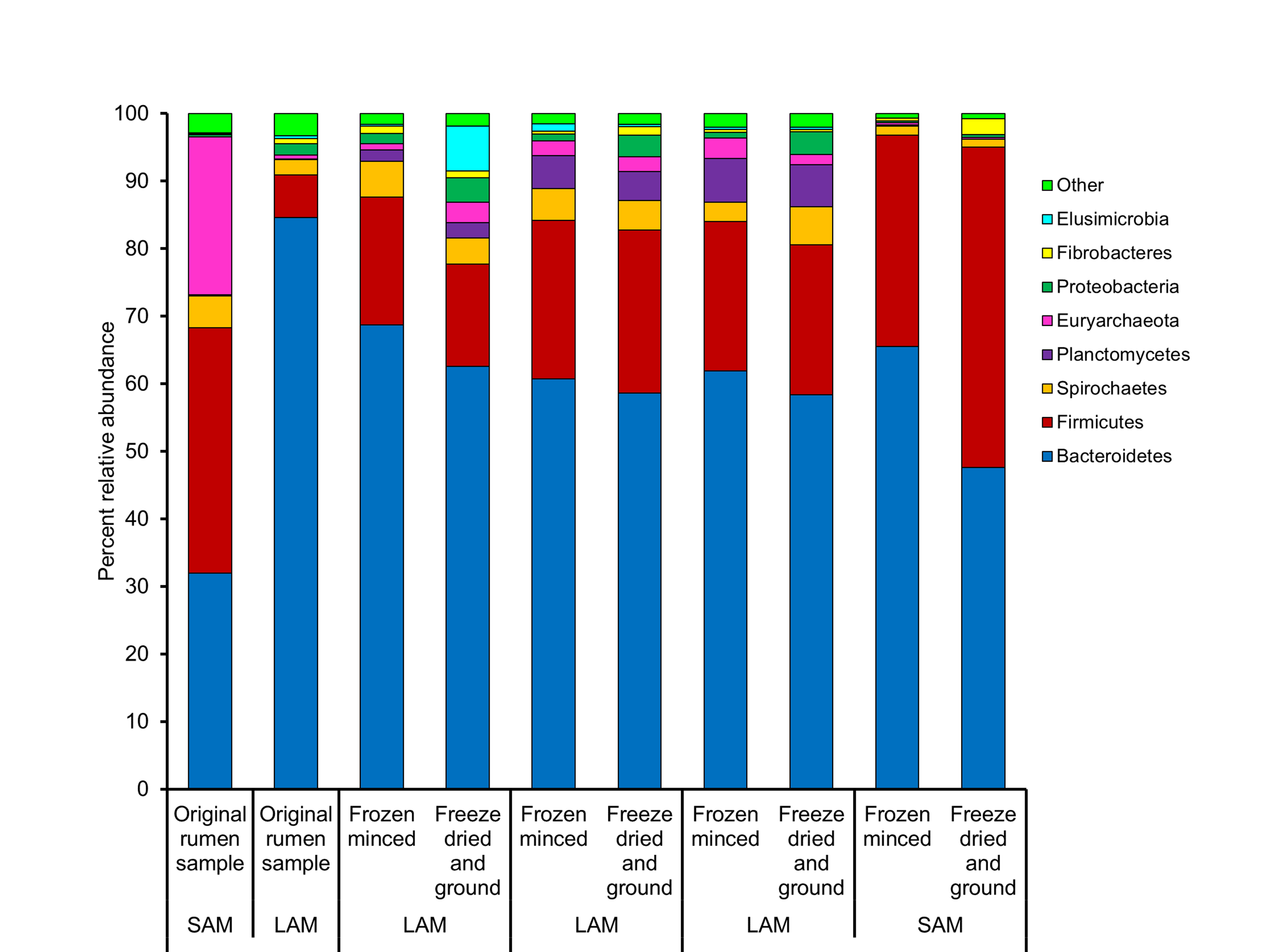

Supplement: FIGURE S1 — The eight most relatively abundant phyla among all samples for each sample type, forage preparation type, and sampling day. LAM, liquid-associated microbes; SAM, solid-associated microbes. [file Image_1.TIF]
