# Supplementary material for: The Type of Forage Substrate Preparation Included as Substrate in a RUSITEC System Affects the Ruminal Microbiota and Fermentation Characteristics
Source: Front Microbiol. 2017 Apr 20;8:704. doi: 10.3389/fmicb.2017.00704 (PMC5397515; doi:10.3389/fmicb.2017.00704)
Supplement: Supplementary file 2 [file Table_1.DOCX]

**Supplementary table S1.** Substrate dry matter content and chemical composition.

|  | Ryegrass | Concentrate |
| --- | --- | --- |
| Dry matter (DM) | 91.9 | 96.4 |
| Neutral detergent fibre (% in DM) | 35.9 | 21.5 |
| Crude protein (% in DM) | 21.8 | 13.7 |
| Crude fat (% in DM) | 4.9 | 5.8 |
| Ash (% in DM) | 11.7 | 6.0 |
